# The role of lung-restricted autoantibodies in the development of primary and chronic graft dysfunction

**DOI:** 10.3389/frtra.2023.1237671

**Published:** 2023-11-09

**Authors:** Wenbin Yang, Emilia Lecuona, Qiang Wu, Xianpeng Liu, Haiying Sun, Hasan Alam, Satish N. Nadig, Ankit Bharat

**Affiliations:** ^1^ Division of Thoracic Surgery, Department of Surgery, Feinberg School of Medicine, Northwestern University, Chicago, IL, United States; ^2^ Division of Trauma & Acute Care Surgery, Department of Surgery, Feinberg School of Medicine, Northwestern University, Chicago, IL, United States; ^3^ Division of Abdominal Transplant, Department of Surgery, Feinberg School of Medicine, Northwestern University, Chicago, IL, United States

**Keywords:** lung-restricted antibodies, primary graft dysfunction, chronic graft dysfunction, *de novo* synthesis, pre-existing auto-antibodies

## Abstract

Lung transplantation is a life-saving treatment for both chronic end-stage lung diseases and acute respiratory distress syndrome, including those caused by infectious agents like COVID-19. Despite its increasing utilization, outcomes post-lung transplantation are worse than other solid organ transplants. Primary graft dysfunction (PGD)—a condition affecting more than half of the recipients post-transplantation—is the chief risk factor for post-operative mortality, transplant-associated multi-organ dysfunction, and long-term graft loss due to chronic rejection. While donor-specific antibodies targeting allogenic human leukocyte antigens have been linked to transplant rejection, the role of recipient's pre-existing immunoglobulin G autoantibodies against lung-restricted self-antigens (LRA), like collagen type V and k-alpha1 tubulin, is less understood in the context of lung transplantation. Recent studies have found an increased risk of PGD development in lung transplant recipients with LRA. This review will synthesize past and ongoing research—utilizing both mouse models and human subjects—aimed at unraveling the mechanisms by which LRA heightens the risk of PGD. Furthermore, it will explore prospective approaches designed to mitigate the impact of LRA on lung transplant patients.

## 1. Introduction

Lung transplantation is a potentially life-saving procedure for patients with end-stage lung diseases who have not responded to other medical treatments. The first successful lung transplantation was performed in 1983, and since then, advances in surgical techniques, organ preservation, and immunosuppressive therapies have improved patient outcomes (1). According to the United Network for Organ Sharing (UNOS), there were 2,692 lung transplants performed in the United States in 2022. The one-year survival rate for lung transplantation is currently around 90%, while the five-year survival rate is approximately 50%, which is significantly worse than other solid transplants. For example, the five-year survival rate for kidney and heart transplantation is approximately 80% and 70%, respectively (https://optn.transplant.hrsa.gov). Primary Graft Dysfunction (PGD) is the predominant risk factor for early mortality including death within 1 year after lung transplantation. However, Chronic Lung Allograft Dysfunction (CLAD) is the leading cause of death beyond 1 year after lung transplantation resulting in an estimated 50% of death (2–4). Importantly, PGD strongly predisposes to the risk of CLAD (5, 6).

Allogeneic immune responses serve as the primary hindrance to pulmonary graft tolerance. Although immunosuppression can prevent T-cell-dependent acute graft rejection, antibody-mediated rejection (AMR), marked by the presence of donor-specific antibodies (DSA), remains an inadequately managed risk factor for CLAD development (7). Recently, non-DSA humoral immune responses have garnered attention in the context of graft rejection (8, 9). In fact, more than one-third of patients with chronic lung disease who undergo lung transplantation have pre-existing antibodies against lung-restricted self-antigens (LRA), such as collagen type V (ColV) and k-alpha1 tubulin (KAT) (5). Interestingly, these antibodies can emerge *de novo* following lung transplantation, playing a role in allograft rejection. LRA have been linked to a host of adverse clinical outcomes, including acute and chronic rejection, as well as increased mortality post-lung transplantation (10–14). This review will delve into the mechanisms through which LRA contribute to the development of lung allograft injury and the prospective strategies designed to diminish the impact of LRA on graft rejection (Table 1).

**Table 1:** LRA in lung transplantation.

	Pre-existing LRA and PGD	*De novo* LRA and CLAD
Development	• IgM somatic hypermutation • Broken B cell anergy by Tregs loss • Gastroesophageal reflux (GER) • Th17-mediated self-reactivity • COPD	• Respiratory viral infection Gastroesophageal reflux (GER) • Tregs loss • Donor-specific antibody (DSA) • IL-17-mediated alloimmunity • Exosomes
Pathogenesis of LRA-inducedlung dysfunction	• LRA extravasation via IL1β increased endothelial permeability • Complement activation • Non-classical monocyte activation via FcγR	• PGD • Complement activation • HIF-1α-mediated upregulation of fibrogenic growth factors • Donor HLA-DR15
Interventions	Anti-C5 antibody IL-1β inhibitor Ex vivo lung perfusion (EVLP)	• Anti-C5 antibody • Anti-IL17 antibody • HIF-1α inhibitor • Exosome inhibitor and • EVLP

PGD, primary graft dysfunction; CLAD, chronic lung allograft dysfunction; IRI, ischemia reperfusion injury.

## 2. Pre-existing LRA development and their role in PGD

### 2.1. Development of LRA prior to transplant

The well-established presence of pre-existing autoantibodies in transplant patients, along with their association with poor clinical outcomes, is evident in multiple solid organ transplants including lung, renal and heart transplants (15, 16). Several hypotheses aim to explain the development of autoantibodies against lung self-antigens (sAgs) in patients prior to lung transplantation. One hypothesis suggests that low-affinity IgM autoantibodies recognize injured and atypically exposed self-proteins as antigenic, acting as templates for somatic hypermutation and class switching to high-affinity IgG/IgA autoantibodies (17, 18).

Generally, newly formed self-reactive B cells in the bone marrow undergo clonal deletion upon binding to sAgs. However, a significant fraction of B cells in the periphery still reacts with sAg but is usually silenced by an immunological tolerance mechanism called anergy. B cells require signals from the BCR and from T cells for activation, and B cell anergy can be broken by CD4+ T helper cells. Hence, another hypothesis for the development of pre-existing LRA is that T cells specific for LRA are not eliminated in the thymus, but are rendered inactive by antigen-specific forkhead box P3 (Foxp3)+ regulatory T cells (Tregs) (19–24). Therefore, loss of Tregs, perhaps due to toxins or infections, could stimulate the expansion of lung tissue-restricted T cells, which in turn could help to break B cell anergy and develop LRA (25–27). These mechanisms also explain the development of pre-existing LRA in patients candidate for transplantation with end-stage lung disease, such as those with COPD or ILD. The end-stage lung disease results in ongoing inflammation and, when coupled with conditions that lead to Treg apoptosis, such as respiratory viral infection frequently seen in lung transplant patients, can result in pre-existing autoimmunity (18).

Additionally, a recent association between gastroesophageal reflux (GER) and the development of LRA has been described (28). The authors demonstrated a strong correlation between pre-lung transplant GER and LRA development. They propose that GER-induced aspiration disrupts fibrils of self-proteins, unmasking Col-V and KAT epitopes, which leads to their immune-histochemical recognition (28).

Finally, as described in the following section, due to their role in *de novo* LRA development, Th17-mediated self-reactivity to ColV is occasionally observed prior to lung transplantation in patients with preexisting pulmonary disease. This phenomenon is typically noted in end-stage lung disease patients who are HLA-DR15+ and are inclined to have lost Treg control (29).

### 2.2. Role of pre-existing LRA in PGD

PGD is a severe form of acute lung injury linked to ischemia-reperfusion injury (occurring within 72 h post-transplantation), representing a significant cause of early morbidity and mortality in the aftermath of lung transplantation. Diagnostically, PGD is characterized by pulmonary edema with diffuse alveolar damage, which clinically manifests as progressive hypoxemia accompanied by radiographic pulmonary infiltrates (30–32). The incidence of PGD (grade 3 or higher) was about 25.7% in a cohort of adult lung transplant recipients in the United States between 2011 and 2018. Furthermore, PGD was associated with an increased mortality rate at one year [OR 1.7 (95% CI 1.2, 2.3), *p* = .0001] (33). PGD has also been linked with a higher risk of CLAD and poorer long-term outcomes (34, 35).

Lung transplant recipients who develop PGD are more likely to have pre-existing LRA than those who do not experience PGD (5, 36). LRA against ColV and KAT are present in over a third of patients undergoing lung transplantation and are associated with a seven-fold increased risk of PGD following transplantation (5). These self-antigens are non-polymorphic, identical across all humans, and part of structural proteins localized to the extravascular space, likely preventing their interaction with circulating LRA during homeostasis (37).

The reason why LRA do not bind to self-antigens in the native lungs before transplantation remains unclear, and interestingly, LRA-mediated injury spares the contralateral native lung in single-lung transplantation models (36). We have recently provided some insights into this question by studying the mechanism of LRA extravasation after lung transplant (36). Utilizing a syngeneic murine model of single lung transplant, we demonstrated that spleen-derived classical monocytes, recruited to the allograft through a CCL2-CCR2 mechanism, secrete IL1β, which heightens vascular permeability by opening endothelial tight junctions via downregulation of the ZO-2 tight junction protein (38). This increased vascular permeability allows LRA to extravasate into the interstitium where they bind to cognate antigens exposed in the transplanted lungs. It is suggested that ischemia-reperfusion injury could reveal epitopes of sequestered self-antigens through increased secretion of matrix metalloproteinases by immune cells, such as neutrophils, leading to presentation of cryptic self-antigens to the immune system (12).

While pre-existing LRA are primarily associated with PGD, recent findings have documented the fast development of *de novo* LRA against ColV. Zaffiri and colleagues demonstrated that ColV is rapidly recognized by B cells, and that the swift seroconversion to anti-ColV antibody is linked to an increased risk of grade 3 PGD in lung transplant recipients (39).

Lastly, although an array of auto-antibodies have been described to develop in patients with COVID-19 (40, 41), none of them seem to belong to the LRA category and thus far, there is no information regarding whether these pre-existing auto-antibodies could affect the development of PGD after lung transplantation (42).

## 3. *De novo* LRA development after lung transplant and their role in CLAD

### 3.1. *De novo* LRA development

Parallel to the mechanism underlying the development of pre-existing LRA, our laboratory has also identified an association between respiratory viral infections, Tregs loss, and the development of *de novo* LRA. We found that lung transplant recipients with microbiologically confirmed respiratory viral infections exhibit decreased numbers of Tregs in the peripheral circulation, of whom approximately 50% develop LRA against ColV and KAT (26). Utilizing animal models, we postulated a “two-hit” model mechanism whereby a combination of Tregs loss and lung injury exposing the sequestered self-antigens would be necessary to induce lung-restricted autoimmunity (25). For instance, we discovered that intra-tracheal administration of hydrochloric acid (simulating GER) in Foxp3-DTR mice only triggered autoimmunity if Tregs were depleted using diphtheria toxin (26). These findings align with our observations that lung transplant patients with GER and diminished Tregs levels manifest *de novo* lung-restricted autoimmunity following transplant (43). Intriguingly, Jonckheere et al. recently demonstrated that Tregs deficiency plays a crucial role in peribronchiolar inflammation, escalating airway permeability, a phenomenon that could facilitate LRA extravasation (44).

A correlation exists between alloimmune responses following lung transplant and autoimmune responses to self-antigens, with DSA development typically preceding that of LRA (45). The theory is that alloimmunity can expose self-antigens or their determinants to the immune system. This occurrence post-transplantation and during calcineurin inhibitor-based immunosuppression, creates a conducive environment for the generation of an immune response against the newly exposed sAgs (46). For example, ColV is released into the transplanted lung after ischemia/reperfusion injury or rejection episodes, which may account for the presence of collagen V–specific T cells isolated from rat lung allografts during rejection (47). Furthermore, Fukami et al. demonstrated that antibodies to donor MHC (DSA) can provoke autoimmunity by developing *de novo* LRA via an IL-17-mediated mechanism (48). In human subjects, it has been suggested that while alloimmunity triggers lung transplant rejection, *de novo* autoimmunity mediated by ColV-specific Th17 cells and monocyte/macrophage accessory cells ultimately leads to progressive airway obliteration (49).

Finally, an additional mechanism for *de novo* LRA development has been described, involving the secretion of donor exosomes (50). Exosomes are small extracellular vesicles secreted by various tissues and are deemed critical mediators of cell-to-cell communication. Recently, the presence of donor human leukocyte antigens and lung sAgs (ColV and KAT) on circulating exosomes released from transplanted lungs was reported (51). Additionally, ColV and KAT are expressed on the surface of exosomes, suggesting that they have the potential to instigate immune responses (52).

### 3.2. Role of *de novo* LRA in CLAD

CLAD is a leading cause of morbidity and mortality among lung transplant recipients and is categorized into two distinct forms: bronchiolitis obliterans syndrome (BOS) and restrictive allograft syndrome (RAS). BOS accounts for approximately 75% of cases, while RAS, although less common, is associated with a poorer prognosis (53, 54). The pathophysiology of CLAD is not fully understood, but several risk factors have been identified, including older age, prior PGD, AMR, cytomegalovirus (CMV) infection, and certain genetic factors. Around 30%–50% of lung transplant recipients develop LRA post-transplantation (55, 56). BOS is a fibroproliferative process characterized by inflammation and progressive fibrosis of the lamina propria, leading to luminal occlusion of small airways, decline in pulmonary function, and ultimately, graft failure. The mechanism by which the *de novo* development of LRA against KAT after lung transplant leads to BOS has been studied recently (11). Tiriveedhi et al. described that the binding of the epithelial gap junction protein KAT with specific LRA leads to HIF-1α-mediated upregulation of fibrogenic growth factors, resulting in increased fibrosis and chronic rejection (57). Similarly, rat studies confirmed that immune responses to ColV induce OB lesions in a lung isograft, but not in the native lung of a syngeneic recipient (37). Patient responses suggest an interaction between Th17 cells and monocytes is critical in the development of BOS (49). This process is exacerbated in patients with a specific donor HLA-DR antigen type, DR15, which confers susceptibility to the development of BOS. HLA-DR15 has the highest ColV peptide binding activity, consistent with high levels of endogenous (Treg-controlled) ColV. Recipients of DR15+ lung transplants who do not possess the DR15 antigen are more likely to develop BOS (29). Apart from LRA against ColV and KAT, other autoantibodies have been associated with the development of CLAD. Conditions of stress, such as ischemia-reperfusion, lead to increased expression of major histocompatibility complex class I chain-related gene A (MICA) in human lung epithelial cells, resulting in apoptosis of these cells and pulmonary fibrosis (58). The development of antibodies against MICA alone, or against both MICA and HLA, is associated with the development of BOS and significantly contributes to the pathogenesis of chronic rejection after lung transplantation (59–61). While not specifically lung-restricted, autoantibodies against angiotensin II type 1 receptor (AT1R) and endothelin-1 type A receptor (ETAR) are also associated with allograft dysfunction (62). These antibodies can activate their target receptors and affect signaling processes. Both AT1R and ETAR stimulation contribute to tissue remodeling and are associated with the development of BOS (62–64).

## 4. Pathogenesis of lung dysfunction by LRA

The mechanisms by which LRAs cause lung allograft injury are not entirely understood, but several hypotheses have been proposed to explain the role of LRAs in the pathogenesis of PGD and CLAD. One of these involves the activation of the complement system. An essential component of the innate immune system, the complement system links innate and adaptive immunity through a combination of soluble and membrane-bound proteins, receptors, and regulators (65). The complement cascade can be activated via the lectin, classical, and/or alternative pathways. These pathways converge at a central amplification step leading to multimeric C3 convertases (65). The lectin complement pathway is activated when mannose-binding lectin (MBL) interacts with carbohydrate motifs, while the classical complement pathway is initiated when C1q binds to the Fc segments of immunoglobulins. The alternative complement pathway is activated by the spontaneous hydrolysis of C3 through complement Factor B (65).

Early studies in lung transplant recipients indicated that patients with PGD after transplantation have higher plasma levels of the complement protein C5a. The use of C1-esterase inhibitor appeared to improve the outcome in cases of severe PGD (PGD3), but these studies did not investigate the presence of pre-existing LRAs (66). More recently, research has shed light on the role of complement activation in PGD when pre-existing LRAs are present (36). Using a mouse model of orthotopic single lung transplant, we found that graft injury in LRA-pretreated mice was associated with activation of both the classical and alternative complement pathways. Interestingly, LRA-induced allograft dysfunction via complement activation appeared to be a distinct process from ischemia-reperfusion injury, as it did not rely on neutrophil recruitment and activation of donor non-classical monocytes, which are known drivers of PGD (36). A study by Patel et al., using a mouse model of chronic obstructive pulmonary disease (COPD), also reported the generation of pre-existing LRAs and activation of the complement system after lung transplantation, leading to PGD (67).

Complement activation also appears to play a role in the development of CLAD. One study reported that induction of obliterative bronchiolitis, a hallmark of CLAD, is partly complement-dependent due to IL-17-mediated downregulation of complement regulatory proteins in the airway epithelium. This was associated with increased levels of C3a, a product of complement activation, in the bronchoalveolar lavage fluid (68).

Additionally, in our recent work, we discovered that donor non-classical monocytes become activated during ischemia-reperfusion through a toll-receptor signaling pathway. Consequently, they produce chemoattractants for neutrophils, which leads to the recruitment of these cells to the transplanted allograft and the development of primary graft dysfunction (PGD) (69, 70). Non-classical monocytes express all activating and inhibitory Fcγ receptors (FcγRI–FcγRIV) on their surface, with FcγRIV (human homolog FcγRIIIA) being the most abundant (71). These receptors play important roles in antibody-dependent cellular cytotoxicity as well as in several autoimmune diseases (72–75). Lung-restricted autoantibodies (LRAs) belong to the IgG family of immunoglobulins, which consist of Fc and F(ab')2 fragments, giving them the ability to activate both the complement pathway and effector cells carrying FcγR (10, 37). Numerous studies suggest that, even with complement activation, FcγRs are significant mediators of IgG effector functions *in vivo* (76). Future research is required to determine if donor non-classical monocytes play a role in LRA-induced PGD.

## 5. Interventions

At our medical center, we screen lung transplant patients for any pre-existing LRAs before the transplantation procedure. If allograft injury occurs, we conduct histological analyses, including complement analyses, on lung parenchymal or transbronchial biopsies. Through prospective analysis of LRAs in 56 patients undergoing lung transplantation, we found that pre-existing LRAs were an independent predictor of grade 3 PGD after lung transplantation. In patients with preexisting LRAs who developed grade 3 PGD, histological features reminiscent of acute antibody-mediated rejection were evident, along with complement (C4d) deposition. Treating these patients with the complement inhibitor eculizumab (a monoclonal antibody targeting complement protein C5), in combination with plasma exchange, helped in resolving lung allograft dysfunction (36). Interestingly, findings in a mouse model suggest that inhibiting IL-1β or the IL-1β receptor may be a therapeutic strategy to prevent both ischemia-reperfusion and LRA-associated injury (36). Notably, canakinumab and anakinra, which are agents that block IL-1β and the IL-1β receptor, respectively, have been approved by the FDA for other indications and have demonstrated relatively benign short-term safety profiles.

Also, a growing medical technique use to assess and putative improve the quality of donor lungs is Ex vivo Lung Perfusion (EVLP), which has been shown to decrease the development of PGD Grade 3 (77). EVLP ameliorates ischemia-reperfusion injury by decreasing donor lung inflammation as well as preserving epithelial integrity (78, 79), which could potentially reduce LRA extravasation and prevent LRA-mediated PGD.

Regarding interventions to prevent the role that LRA have in CLAD development work from Tiriveedhi and colleagues showed that neutralization of IL-17 with a blocking antibody in mouse showed significant decrease in the histological markers of obliterative airway lesions and decrease in antibodies to sAgs (80). Also, it has been postulated that the use of EVLP to treat the donor lung with inhibitors of exosome release could prevent allograft immune responses to ColV and KAT (9).

## 6. Conclusion

Despite the improvements in surgical techniques and immunosuppression regimes, lung transplant is still behind the success of other solid organ transplants. The presence of LRA before and/or after lung transplant provides another layer of complexity. Over the last decades, there has been growing scientific research on autoimmune responses in allograft rejection, in both cellular and humoral immunology. Strong evidence suggests that LRA is linked to an increased risk of PGD and CLAD following lung transplantation. However, determining the most effective treatment approach remains uncertain. Complement and IL1β inhibition are hopeful treatments that could be foreseen in the future, nevertheless, the pace of advancement in clinical trials, particularly randomized controlled trials, has been slow. The gap between basic research and clinical trials has impeded the translation of experimental discoveries into enhanced clinical protocols and improved patient outcomes. Until recently, the absence of readily available commercial assays for identifying LRA posed a substantial challenge in managing autoimmunity in lung transplantation. Fortunately, several commercial assay kits for detecting antibodies against COLV, KAT, AT1R, and ETAR are now accessible, potentially bolstering our capacity to detect autoimmune responses in allograft rejection. In conclusion, the continuation of the success in recent basic and translational research and the incorporation of the detection and management of LRA into clinical practice has the potential to improve lung transplant survival.
